# Hormonal and transcriptional analyses provides new insights into the molecular mechanisms underlying root thickening and isoflavonoid biosynthesis in *Callerya speciosa* (Champ. ex Benth.) Schot

**DOI:** 10.1038/s41598-020-76633-x

**Published:** 2021-01-08

**Authors:** Shaochang Yao, Zuzai Lan, Rongshao Huang, Yong Tan, Ding Huang, Jinyuan Gu, Chunliu Pan

**Affiliations:** 1 College of Pharmacy, Guangxi University of Chinese Medicine, Nanning, Guangxi People’s Republic of China; 2 Guangxi Botanical Garden of Medicinal Plants, Nanning, Guangxi People’s Republic of China

**Keywords:** Developmental biology, Molecular biology

## Abstract

*Callerya speciosa* (Champ. ex Benth.) Schot is a traditional Chinese medicine characterized by tuberous roots as the main organ of isoflavonoid accumulation. Root thickening and isoflavonoid accumulation are two major factors for yield and quality of *C. speciosa*. However, the underlying mechanisms of root thickening and isoflavonoid biosynthesis have not yet been elucidated. Here, integrated morphological, hormonal and transcriptomic analyses of *C. speciosa* tuberous roots at four different ages (6, 12, 18, 30 months after germination) were performed. The growth cycle of *C. speciosa* could be divided into three stages: initiation, rapid-thickening and stable-thickening stage, which cued by the activity of vascular cambia. Endogenous changes in phytohormones were associated with developmental changes during root thickening. Jasmonic acid might be linked to the initial development of tuberous roots. Abscisic acid seemed to be essential for tuber maturation, whereas IAA, *cis*-zeatin and gibberellin 3 were considered essential for rapid thickening of tuberous roots. A total of 4337 differentially expressed genes (DEGs) were identified during root thickening, including 15 DEGs participated in isoflavonoid biosynthesis, and 153 DEGs involved in starch/sucrose metabolism, hormonal signaling, transcriptional regulation and cell wall metabolism. A hypothetical model of genetic regulation associated with root thickening and isoflavonoid biosynthesis in *C. speciosa* is proposed, which will help in understanding the underlying mechanisms of tuberous root formation and isoflavonoid biosynthesis.

## Introduction

*Callerya speciosa* (Champ. ex Benth.) Schot, a perennial shrub plant in the *Fabaceae* family, is widely cultivated in South China and commonly used for medicine. Tuberous root is the medicinal organ of *C. speciosa*, which originates from the expansion of fibrous roots. Tuber morphogenesis, along with the accumulation of a large amount of starch as well as health-promoting components, is the main processes of tuberous root development^1^. Isoflavonoids as the main medicinal compounds have been produced in the tuberous roots of *C. speciosa*, such as the index compounds maackiain and formononetin^2^, which have made considerable contributions to immunity enhancement, hepatoprotection, arresting cough, and expectorant and anti-asthmatic effects^3^. The quality of *C. speciosa* is mainly evaluated according to the degree of root thickening and the content of isoflavonoids, while the harvest period is primarily depended on the yield. Generally, the fibrous roots of *C. speciosa* possess the potential to form tuberous roots, but limited fibrous roots can transfer into tuberous roots^4^. Studies of root thickening and isoflavonoid biosynthesis of *C. speciosa* are scarce.

Tuber morphogenesis is mainly regulated by the interaction between primary and secondary cambia. The primary cambia usually determine the formation of root/stem, while the secondary cambia affect root/stem thickening by cell division and expansion^5^. The formation of tuber usually involves three common stages: initiation stage, rapid-thickening stage and stable-thickening stage^6^, which is determined mainly by endogenous and environmental factors, such as photoperiod, high sucrose, and water supply^7–9^. Great efforts have been made to explore the roles of endogenous phytohormones in the initiation and development of tuberous roots. The synergistic actions of various phytohormones, such as auxins, abscisic acid (ABA), gibberellins (GAs), ethylene (ETH), jasmonic acid (JA) and cytokinins (CKs), finally result in the bulking of tuberous roots^10,11^. Tuber morphogenesis is a complex biological process involving many specific genes and proteins. Transcriptome techniques have contributed to our understanding of these genes involved in regulation tuberous root formation. In medicinal plant *Rehmannia glutinosa*, transcriptome analysis indicated that 6032 DEGs related to hormone signaling, signal transduction and light signaling were identified during tuberous root formation^5^. In potato transcriptomic data, some of these genes were involved in plant hormone signal transduction, with GID1-like GA receptor (*StGID1*) being up-regulated^12^. In sweet potato, several transcriptome analyses revealed that a large number of genes were highly regulated during the storage root formation, which participated in starch and lignin synthesis, cell division, and expansion^10,13^. In addition, 191 DEGs were found to be involved in functions such as plant growth and development, metabolism, cell organization and biogenesis, signal sensing and transduction, and plant defense response in radish^14^. Sucrose content dominantly decreased at an early stage of bulb development in onion, whereas the fructose and glucose contents increased significantly at the mature stage, suggesting that sucrose metabolism plays an important role in onion bulb formation^15^. The previous studies focused mainly on crops, but the root expansion process in medicinal plants may be different from that in crops. Little is known about the genetic regulation of the initiation and development of tuberous roots in *C. speciosa*.

As a branch of the flavonoid pathway, the biosynthesis of isoflavonoids shares the common upstream pathway with flavonoid biosynthesis under the actions of phenylalanine ammonialyase (PAL), cinnamate 4-hydroxylase (C4H), 4-coumarate-CoA ligase (4CL), chalcone synthase (CHS), chalcone isomerase (CHI), and chalcone reductase (CHR)^16^. In the downstream pathway, isoflavonoids are synthesized from liquiritigenin under the catalysis by 2-hydroxyisoflavanone dehydratase (HIDM) and isoflavone synthase (IFS), and then modified by various tailoring processes including hydroxylation, methylation, and glycosylation under the actions of other enzymes, such as isoflavone 4′-*O*-methyltransferase (HI4′OMT), vestitone reductase (VR), and cytochrome P450 members^17^. The gene expression analysis of isoflavonoid biosynthesis has been studied by transcriptome analysis in several legume plant species, such as soybean, *Pueraria candollei* and *P. lobata*^16,18,19^. However, many genes encoding key enzymes in the isoflavonoid pathway are still not well defined, especially the genes encoding downstream pathway enzymes. A comprehensive understanding of the genes involved in isoflavonoid biosynthesis during the development of tuberous roots in *C. speciosa* will help in revealing the mechanism of isoflavonoid biosynthesis.

In this study, twelve libraries prepared from four different age points during *C. speciosa* tuber expansion were sequenced by using BGISEQ-500 platform. A detailed comparative mRNA analysis was detected, and the key genes that potentially participated in root thickening and isoflavonoid biosynthesis were identified by integrating the phenotypic data and gene expression profiles. The results will be valuable for further research, and will help in understanding the molecular mechanisms of tuberous root formation and isoflavonoid metabolism in *C. speciosa*.

## Results

### Definition of key developmental stages of tuberous roots

**Figure 1:**
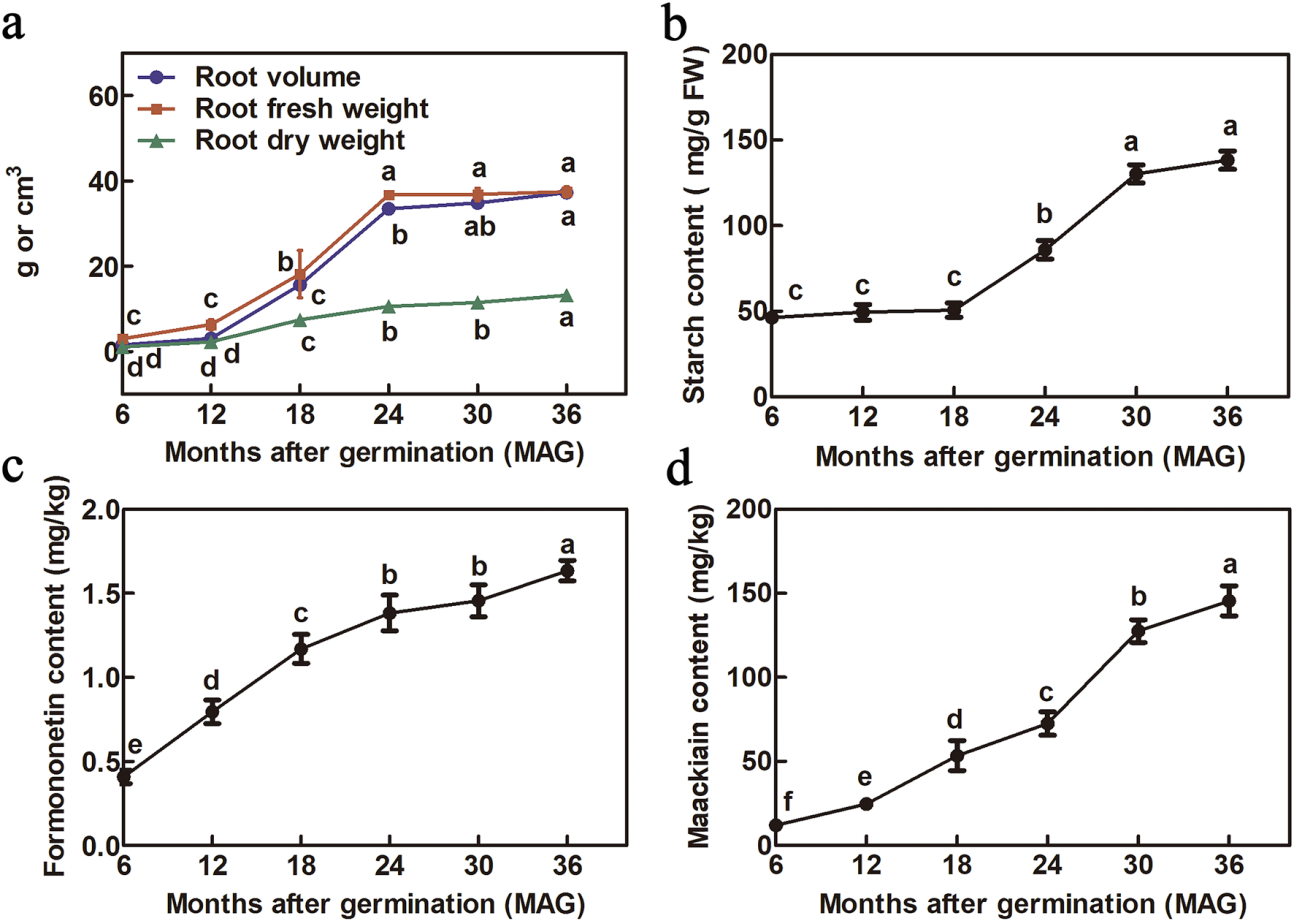
Determination of growth indices of *C. speciosa* roots at different developmental ages. (**a**) Root volume, fresh weight and dry weight. (**b**) Starch content. (**c**) Formononetin content. (**d**) Maackiain content. All data shown reflect the average mean of three biological replicates (*n* = 3). Lowercase letters represent the significance test results at 0.05 level.

To determine the key developmental stages of tuberous roots, roots were sampled at half-year intervals after germination. The root volume, fresh weight and dry weight increased slowly in 6–12 months after germination (MAG), but they increased significantly from 12 to 24 MAG. From 24 to 36 MAG, the root volume and fresh weight were barely changing, while the root dry weight was increasing (Fig. 1a). Thus, the rapid growth phase occurred by root saturation with water. The starch content analysis revealed that it increased significantly from 18 to 30 MAG, but there was no significant difference before 18 MAG (Fig. 1b). However, the formononetin and maackiain contents increased continuously along with the process of tuberous root development (Fig. 1c,d), which indicated that formononetin and maackiain accumulation simultaneously increased along with dry weight during the growth of *C. speciosa*.

**Figure 2:**
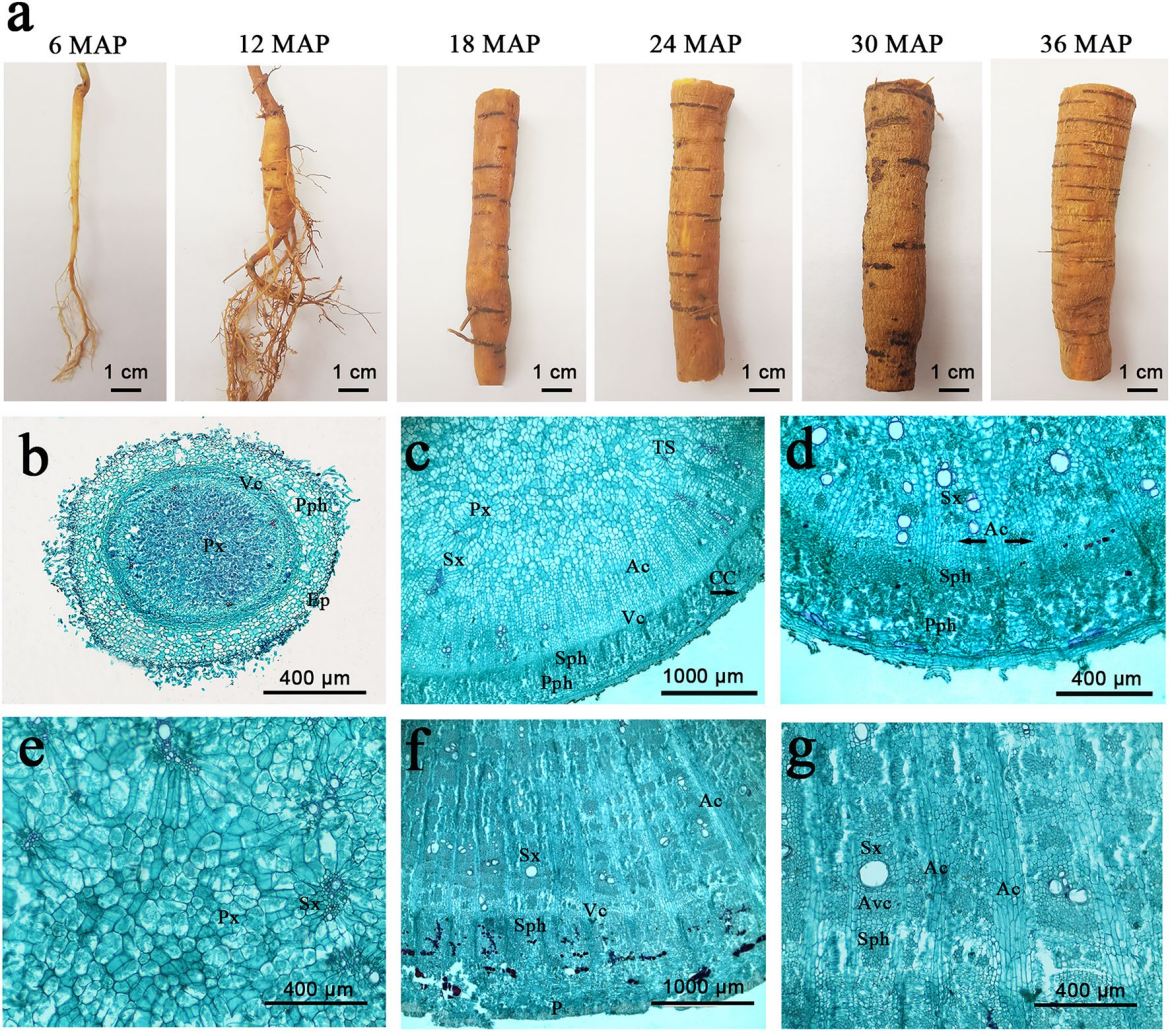
Morphological and anatomical features of *C. speciosa* roots at different developmental ages. (**a**) Root morphology. (**b**) Transverse section of root at 6 MAG. (**c**) Transverse section of root at 12 MAG. (**d**,**e**) Transverse section of root at 18 MAG. (**f**,**g**) Transverse section of root at 24 MAG. *Ac* anomalous cambium, *Avc* accessory cambium, *CC* cork cambium, *Ep* epidermis, *P* periderm, *Pph* primary phloem, *Px* primary xylem, *Sph* secondary phloem, *Sx* secondary xylem, *TS* the scattered tertiary structure, *Vc* vascular cambia.

Morphological and anatomical analyses were also performed to reveal the secondary meristem activity during the tuberous root thickening. The morphological analysis revealed distinct phenotypic characteristics appeared at different developmental ages. The tuberous root was markedly enlarged from 12 MAG, suggesting that the secondary meristem activity might be activated before that phase (Fig. 2a). The anatomical results also showed that the vascular cambia (Vc), which cued fibrous roots to tuberous roots, appeared before 6 MAG. Thus, the 6 MAG represented a critical point in the transition from fibrous roots to tuberous roots, in which vascular bundles were well developed and secondary cambia initiated (Fig. 2b). After the vascular cambia appeared, several secondary structures, including secondary xylem (Sx) and secondary phloem (Sph), anomalous cambia (Ac), and the scattered tertiary structures (TS), which promoted root thickening, observed well-developed at 12 MAG and secondary cambia divided continuously at 18 MAG (Fig. 2c,d). Due to the meristematic cells proliferated continuously, secondary phloem cells outward and secondary xylem cells inward were produced, resulting in the secondary xylem much larger than the phloem (Fig. 2e,f). From 24 MAG, accessory cambium (Avc) appeared in the secondary phloem, and the continuous division of these cambium cells leaded to rapid thickening of roots (Fig. 2f,g). The thickening rate of roots slowed down from 30 MAG and tended to stabilize. Overall, the growth cycle of *C. speciosa* could be divided into three stages: initiation, rapid-thickening and stable-thickening stage. To explore the molecular mechanism during root thickening, four samples were selected for further research, including initiated thickening stage (6 MAG), early-rapid thickening stage (12 MAG), mid-rapid thickening stage (18 MAG), and stable thickening stage (30 MAG).

### Overview of de novo transcriptome assembly

Approximately 799.94 Mb original data in total were obtained using BGISEQ-500 platform. After filtering low-quality reads and adaptor sequences, 78.23 Gb clean reads were obtained and processed by de novo analysis using Trinity software. Then, Tgicl software was used on unigenes to remove abundance, and 153,153 unigenes were obtained. The N50, Q30 and gene mapped percentage was 2167 bp, 89.14%, and 88.09%, respectively (Supplementary Table S1). The length distribution of all the assembled transcripts shown in Supplementary Fig. S1a, which indicated that 24.93% of the unigenes were longer than 2000 bp. Functional annotation revealed that 106,882 (69.79%), 100,406 (65.56%), 79,007 (51.59%), 86,688 (56.60%), 86,701 (56.61%), 80,297 (52.43%), 75,729 (49.45%), 45,329 (29.60%) were aligned with eight databases (NR, NT, SwissProt, KEGG, KOG, Pfam, GO, and InterPro) respectively (Supplementary Fig. S1b). Furthermore, the similarity distribution indicated that there were 21.30% of all the unigenes mapped to *Cicer arietinum* by aligning to NR database according to their amino acid sequences, followed by *Cajanus cajan* (15.96%), *Glycine max* (14.56%), belonged to the same Leguminosae family (Supplementary Fig. S1c).

KOG is a database of orthologous gene families, in which 86,701 unigenes were classified to 25 functional classifications. The “signal transduction mechanisms” (13,227 unigenes) were annotated in KOG database (Supplementary Fig. S1d). Moreover, 81,412 unigenes were involved in the KEGG pathways, which were categorized into five functional groups, including metabolism (58.77%), organismal systems (23.64%), environmental information processing (5.15%), genetic information processing and cellular processing (4.99%) (Supplementary Fig. S1e). Carbohydrate metabolism was identified to be the most important pathway during tuberous root development, such as starch and sucrose metabolism, amino sugar and nucleotide sugar metabolism (Supplementary Fig. S1f), while phenylpropanoid biosynthesis was the dominated pathway in biosynthesis of other secondary metabolites (Supplementary Fig. S1g).

**Figure 3:**
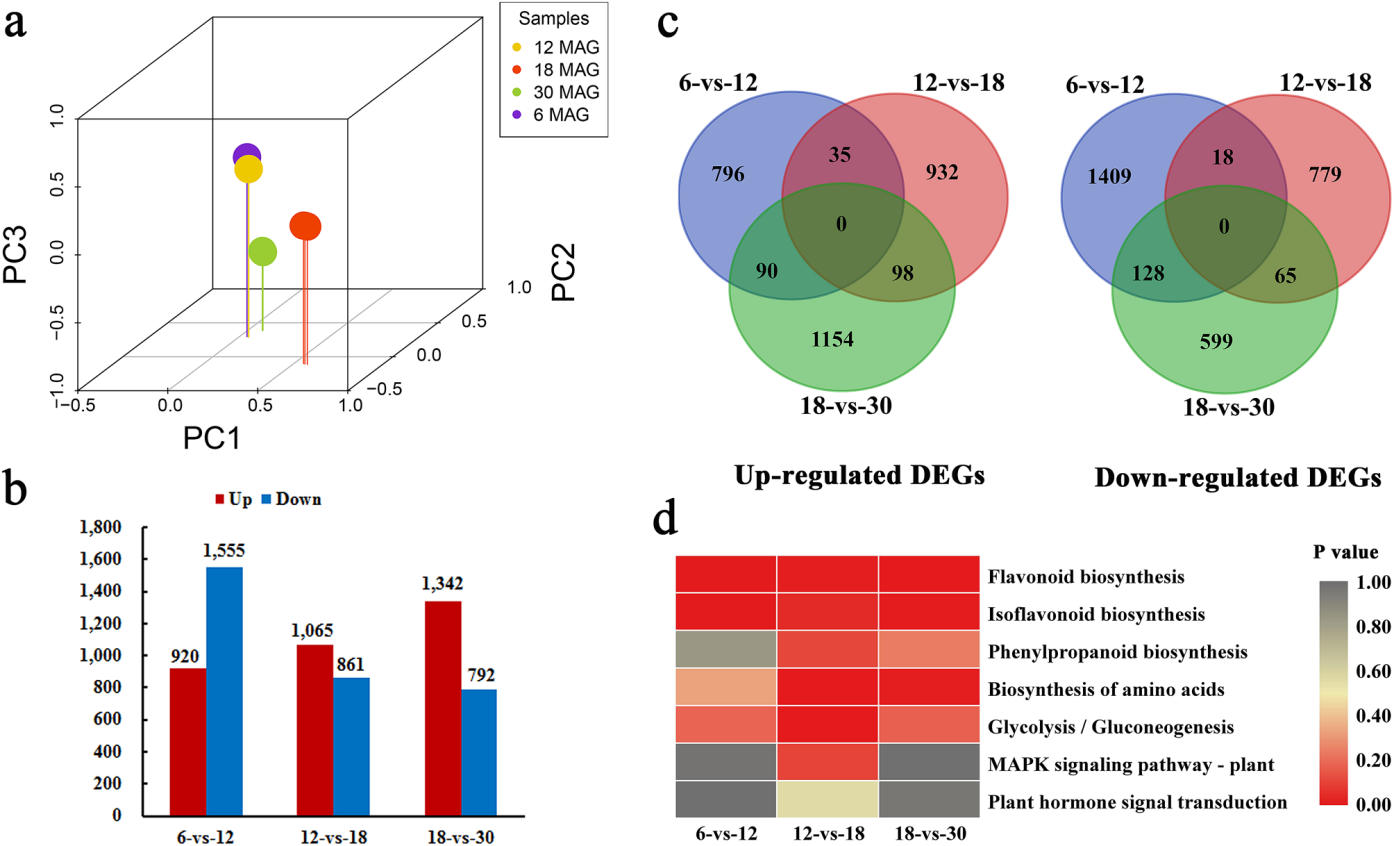
Global gene expression profiling of *C. speciosa* roots at different developmental ages. (**a**) PCA analysis of the RNA-Seq data. (**b**) Numbers of identified differentially expressed genes (DEGs) in the sequential pairwise comparisons. (**c**) Venn diagrams of DEGs among the three comparisons. (**d**) Significantly enriched KEGG pathways of DEGs. The numbers (6, 12, 18, 30) represent the four different developmental ages.

Principal component analysis (PCA) showed that the 12 samples could be clearly assigned to four groups. There was a significant difference between 18 and 30 MAG, while 6 MAG and 12 MAG clustered together, which indicated that the transcriptomic profiles were similar between initiated thickening and early-rapid thickening stages while those of mid-rapid thickening and stable thickening stages were distinct (Fig. 3a).

### Identification of differentially expressed genes (DEGs) in transcriptome for *C. speciosa*

A total of 4337 DEGs were identified among three pairwise comparisons: 2, 475 DEGs in 6-vs-12, 1926 DEGs in 12-vs-18, and 2134 DEGs in 18-vs-30. More down-regulated DEGs appeared in 6-vs-12 comparison, whereas the opposite occurred in 12-vs-18 and 18-vs-30 comparisons (Fig. 3b). A complete list of the detected DEGs showed detailly in Supplementary Table S2. The Venn diagram of DEGs showed that none were commonly up- or down-regulated in all the pairwise comparisons, implying that the DEGs might be regulated in certain period (Fig. 3c). The clustering analysis of DEGs were also performed by K-means clustering method based on similarities in gene expression profiles. The results showed that DEGs were mainly divided into six clusters, in which 379 genes were clustered in the up-regulated subclass and 820 genes were highly expressed at the rapid-thickening stages (Supplementary Fig. S2).

For better comprehension of DEGs functions, 48 GO categories were identified. For biological processes, DEGs were involved mainly in “metabolic process” and “cellular process” (Supplementary Fig. S3). Functional analysis by KEGG revealed that DEGs were enriched predominantly in “flavonoid biosynthesis”, “isoflavonoid biosynthesis”, “glycolysis/gluconeogenesis”, and “phenylpropanoid biosynthesis” pathways in the three pairwise comparisons (Supplementary Fig. S4). In particular, “flavonoid biosynthesis” and “isoflavonoid biosynthesis” pathways were significantly enriched in all pairwise comparisons. The “biosynthesis of amino acids” pathway were significantly enriched in two comparisons (12-vs-18 and 18-vs-30), whereas “glycolysis/gluconeogenesis”, “MAPK signaling pathway”, and “plant hormone signal transduction” showed significant difference in 12-vs-18 comparison (Fig. 3d). These DEGs might be closely related to tuberous root development and isoflavonoid metabolism in *C. speciosa*.

### Endogenous hormone contents and DEGs related to hormone signaling

**Figure 4:**
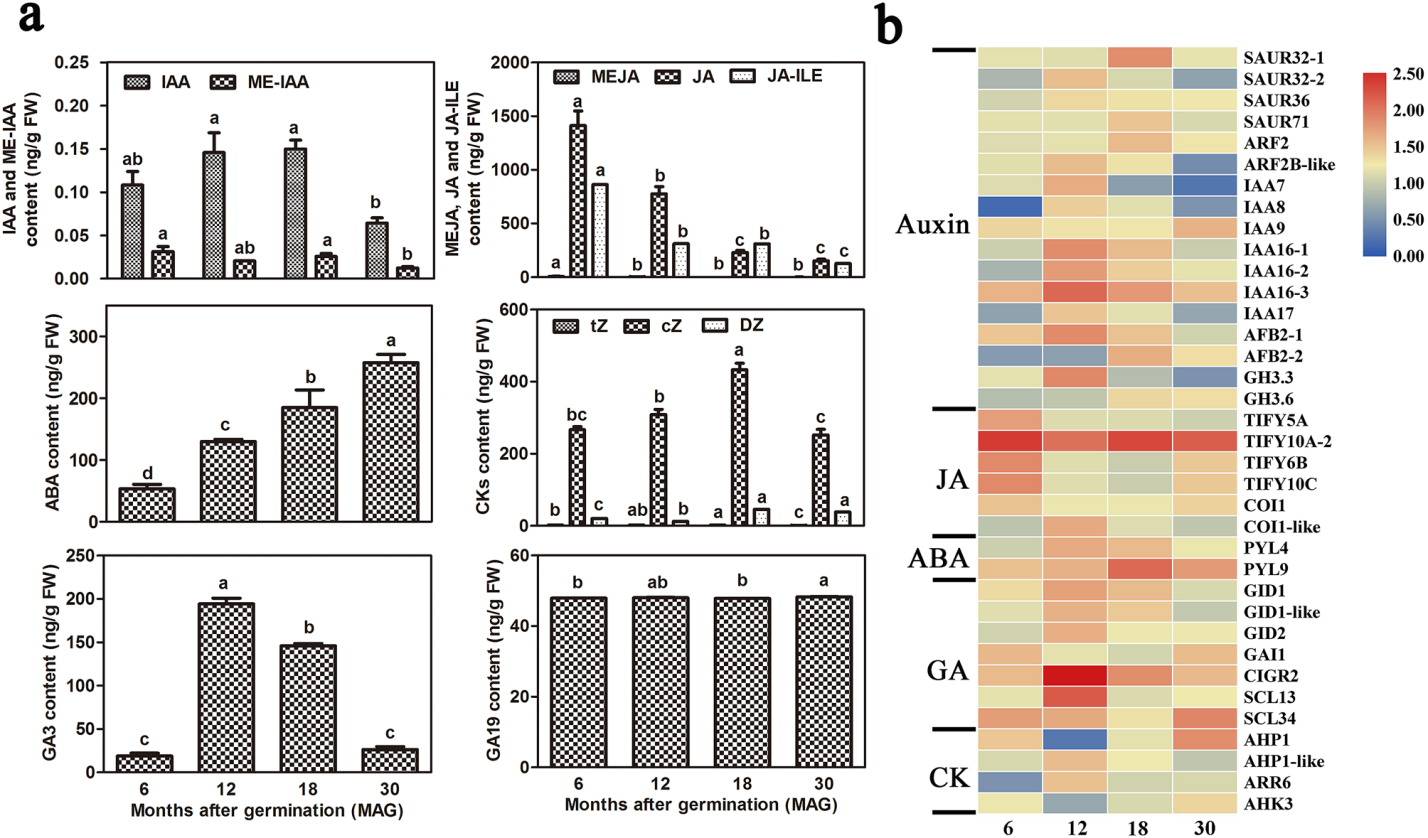
Trends in endogenous phytohormone contents (**a**) and expression profiles of hormone related DEGs (**b**) in *C. speciosa* roots. (**a**) Trends in endogenous phytohormone contents. (**b**) The expression profiles of genes encoding hormone signaling. Heat map indicates the log_10_-transformed FPKM expression values. Changes in expression level are indicated by a change in color; from blue to red indicates an expression level from low to high. All data shown reflect the mean of three biological replicates (*n* = 3). Means with different letters in each sample represent a significant difference at 0.05 level.

To investigate the roles of endogenous hormones in root thickening, we analyzed the auxins, CKs, GAs, JA, and ABA contents in *C. speciosa* roots by UPLC–MS/MS in the present study (Fig. 4a). The results showed that both IAA and GA_3_ contents were significantly high at the rapid thickening of tuberous roots, suggesting that high IAA and GA_3_ levels might promote tuber enlargement. Similarly, *cis*-zeatin (*c*Z) as the main cytokinin in *C. speciosa* tuberous roots, peaked at 18 MAG. By contrast, the highest JA content appeared at 6 MAG, corresponding to the initiation development of tuberous roots, whereas the opposite trend for ABA content was observed at the same age point. GA_19_ content maintained at a relatively constant level during root thickening. In the corresponding hormone signaling pathways, 36 DEGs were identified among 4337 DEGs (Supplementary Table S3). In auxin transduction pathway, the expression levels of genes encoding AUX/IAA auxin-responsive protein (*IAA16s* and *IAA17*), auxin signaling F-box 2 (*AFB2-1*) and auxin-responsive protein *GH3.3* were higher at 12 MAG, while that of small auxin up RNA (*SAUR32-1*, *SAUR71*), auxin response factor (*ARF2*), and *AFB2-2* were highly expressed at 18 MAG. The genes encoding JA ZIM-domain protein (*TIFYs*) and receptor *COI1* showed high expression levels at the initiated stage (6 MAG), whereas two ABA receptor *PYL* genes were highly expressed at the rapid expansion stage (12 and 18 MAG). Most of the DEGs related to GA and CK signaling pathways were highly expressed at the early-rapid expansion stage (12 MAG), such as gene encoding receptor *GIDs*, chitin-inducible gibberellin-responsive protein 2 (*CIGR2*), scarecrow-like 13 (*SCL13*), histidine-containing phosphotransfer protein 1-like (*AHP1-like*) and cytokinin response regulator (*ARR6*) (Fig. 4b). These results were consistent with those of endogenous hormones, and indicated that these genes might be involved in regulating the hormonal signal transduction during root thickening.

### DEGs related to sucrose and starch metabolism

**Figure 5:**
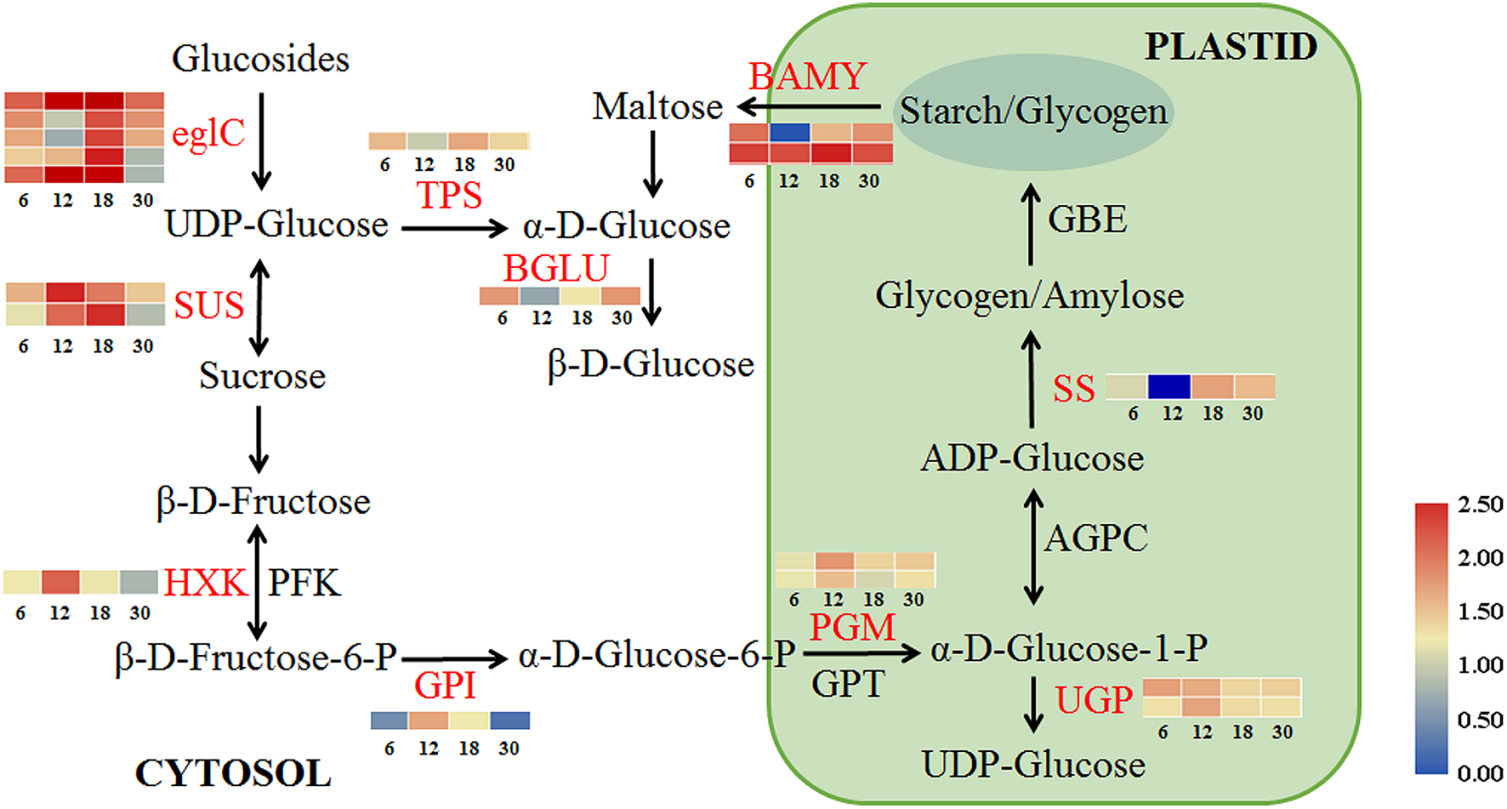
Expression profiles of DEGs related to sucrose and starch metabolic pathway during *C. speciosa* root thickening. Heat maps indicate the log_10_-transformed FPKM expression values. The numbers (6, 12, 18, 30) at the bottom of the heat maps represent the four different developmental ages. Changes in expression level are indicated by a change in color; from blue to red indicates an expression level from low to high. All data shown reflect the mean of three biological replicates (*n* = 3).

The starch content increased significantly from 18 to 30 MAG (Fig. 1b). In the sucrose and starch metabolism pathway, key enzymes maintain the metabolic balance of glucose, fructose, sucrose, and starch/glycogen. Among the 4337 DEGs, 18 genes encoding key enzymes in the sucrose and starch metabolism pathway were identified (Supplementary Table S3). Heatmap analysis revealed that the expression profiles of 18 DEGs obviously differed among the four samples. The expression of gene encoding granule-bound starch synthase 1 (*GBSS1*) was higher at 18 and 30 MAG, whereas genes encoding sucrose synthase (*SUSs*) were highly expressed at 12 and 18 MAG. Genes encoding phosphoglucomutase (*PGM*), glucose-6-phosphate isomerase (*GPI*), UTP–glucose-1-phosphate uridylyltransferase (*UGP*), and hexokinase 1 (*HXK1*) were highly expressed at 12 MAG. Two genes encoding glucan endo-1, 3-beta-glucosidase (*eglC*) were highly expressed at both 12 MAG and 18 MAG, while the expression of other three genes in the same family were at high levels at 18 MAG. In addition, the expression of two genes encoding beta-amylase (*BAMY*) differed among different developmental ages of roots, and *BAM9* were at a higher level than *BAM1* (Fig. 5). Therefore, sugar metabolism pathway might be activated by these metabolic related DEGs at rapid-thickening stage of *C. speciosa*, but the DEGs associated with starch metabolism pathway might play important roles in promoting starch accumulation at stable thickening stage, which was consistent with the trend of starch content.

### DEGs related to isoflavonoid biosynthesis

**Figure 6:**
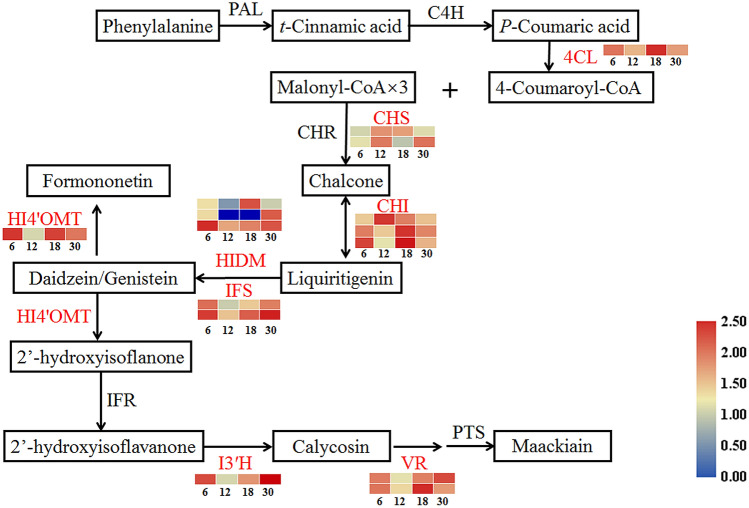
Expression profiles of DEGs involved in isoflavonoid metabolic pathway during *C. speciosa* root thickening. The log_10_-transformed FPKM expression values are shown. The numbers (6, 12, 18, 30) at the bottom of the heat maps represent the four different developmental ages. Changes in expression level are indicated by a change in color; from blue to red indicates an expression level from low to high. All data shown reflect the mean of three biological replicates (*n* = 3).

The content change of formononetin and maackiain increased gradually during the tuberous root development (Fig. 1c,d). Analysis of the *C. speciosa* transcriptomic data allowed us to identify 15 genes involved in isoflavonoid biosynthesis among 4337 DEGs, including genes encoding 4-coumarate: CoA ligase (*4CL*), *O*-methyltransferase (*HI4′OMT*), vestitone reductases (*VRs*), 2-Hydroxyisoflavanone dehydratases (*HIDMs*), chalcone synthases (*CHSs*), chalcone-flavonone isomerases (*CHIs*), isoflavone synthases (*IFSs*), and isoflavone-3′-hydroxylase (*I3′H*) (Supplementary Table S3). These DEGs had different expression patterns among the four samples. Two *CHSs* (*CHS1* and *CHS7*) were both highly expressed at 12 MAG. Like *CHSs*, *CHI3* also had highly expression at 12 MAG, but more DEGs showed a low expression level at 12 MAG, such as *4CL*, *IFSs, I3′H*, *VRs*, and *HI4′OMT* (Fig. 6). Hence, we speculated that isoflavonoid biosynthesis might be promoted under the complex synergistic effects of these genes during the thickening of *C. speciosa* tuberous roots.

### DEGs related to MAPK and calcium signaling

The regulation of genes associated with mitogen-activated protein kinase (MAPK) and calcium signaling were also identified from 4337 DEGs in the present study (Supplementary Table S4). Four MAPK signaling-related genes were annotated, including *MPK3*, *MPK7*, *MPK9*, and *MAPKKK18*. Of these, three genes (*MPK3*, *MPK9* and *MAPKKK18*) were highly expressed at the early-rapid thickening stage (12 MAG). Calcium-regulated transduction is another key signaling transduction pathway in cell development. A total of 20 DEGs were homologous with calcium signal-related genes, including nine calcium binding protein transcripts (*CBPs*), four calreticulin transcripts (*CBLs*), three calcium-transporting ATPase transcripts (*CTAs*), two calcium-dependent protein kinase transcripts (*CDPKs*), and two calcium exchanger transcripts (*NCLs*). It is worth noticing that most of these genes were highly expressed at the initial thickening stage (6 MAG) and early-rapid thickening stage (12 MAG), indicating that genes encoding the calcium-regulated signaling pathway might play vital roles in initiation of root thickening in *C. speciosa*.

### DEGs related to cell wall and cell cycle

Among 4337 DEGs, a total of 34 transcripts associated with cell expansion, cell wall synthesis, cell cytoskeleton and cell cycle regulation were identified during the expansion of tuberous roots, including transcripts encoding xyloglucan endotransglucosylase/hydrolase (*XTH*), expansin (*EXP*), extension (*EXT*), pectinesterase (*PE*), fructokinase (*FRK*), cyclin-dependent kinases G-2 (*CDKG-2*), tubulin beta-2 chain-like (*TUB2*), cell division cycle protein 48 (*CDC48*), and cyclin-dependent kinases regulatory subunit (*CKS1*) (Supplementary Table S5). Most of the genes related to cell expansion, cell wall synthesis, and cell cytoskeleton were highly expressed at the rapid thickening stages (12 and 18 MAG), whereas the genes related to cell cycle regulation showed the opposite trend.

### Differentially expressed transcription factors (TFs)

A total of 230 TF-encoding genes belonging to 30 TF families were identified from 4337 DEGs during root thickening (Supplementary Table S2). Among them, 41 DEGs belonging to AP2/ERF, MYB, NAC, MADS, bHLH, and WRKY families have previously implicated in plant growth and development (Supplementary Fig. S5). AP2/ERF TF is one of the largest superfamilies in plants, indicating that different members might play diverse roles in various root thickening stages. The majority of *ERFs* transcripts were highly expressed at 12 MAG and 18 MAG, especially *ERF109-like*, *ERF105* and *ERF17-like*. Genes belonging to MYB, NAC, and WRKY families were highly expressed at the rapid expansion stage (12 and 18 MAG), whereas 4 *bHLHs* and 2 *MADSs* were highly expressed at 18 MAG. Our results suggested that these TFs might potentially participate in the developmental regulation of *C. speciosa* tuberous root thickening and the transcriptional regulation of isoflavonoid biosynthesis.

### Validation of candidate DEGs by qRT-PCR analysis

**Figure 7:**
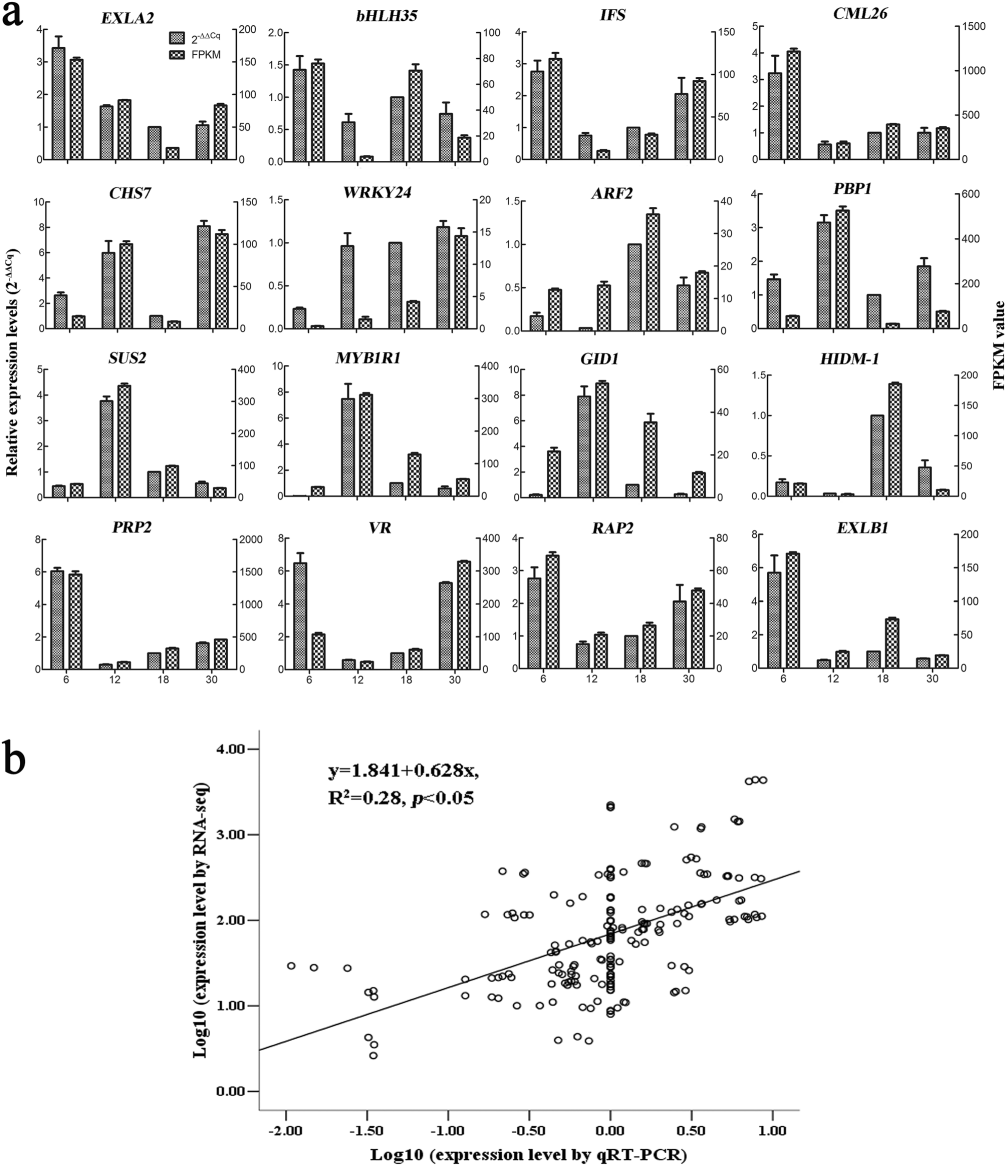
Validation of the transcriptomic data with qRT-PCR. (**a**) Comparison of the expression levels between qRT-PCR and FPKM values identified by transcriptome. (**b**) Correlation plot of the qRT-PCR (2^−ΔΔCq^) and FPKM values. The *R*^2^ value represents the correlation between the qPCR and RNA-seq results.

To validate the reliability of the transcriptomic data, 16 DEGs were selected randomly and their relative expression levels were determined by quantitative real-time PCR (qRT-PCR) analysis (Supplementary Table S6). The expression levels of the selected genes were calculated using 2^−ΔΔCq^ method. We compared the expression data between RNA-seq and qRT-PCR, and the correlation was calculated using log_10_ fold variation measurements to produce a scatter plot. As shown in Fig. 7a, the relative expression levels (2^−ΔΔCq^) of all the selected genes were significantly consistent with the expression levels determined using the transcriptome data (FPKM values). The Pearson correlation values (*R*^2^) between the relative expression levels and FPKM values were 0.28 (*p* < 0.05) (Fig. 7b). Thus, the qRT-PCR analysis confirmed the validity of the transcriptome data, indicating that the transcriptome data were reliable and accurate.

## Discussion

*Callerya speciosa* (Champ. ex Benth.) Schot is a traditional Chinese medicine characterized by tuberous roots as the main organ of isoflavonoid accumulation. The formation and development of tuberous roots is a complex and genetically programmed process that involves the proliferation of secondary meristems and the accumulation of carbohydrates and secondary metabolites^5^. Based on several growth and content indexes determination in this study, roots saturation with more water occurred at the rapid growth phase, while the content of carbohydrates and secondary metabolites increased at the later period of root thickening (Fig. 1). Morphological and anatomical analyses verified that vascular cambia cued fibrous roots to tuberous roots in *C. speciosa*, which was already well formed at 6 MAG. Secondary xylem and phloem differentiated under the activity of vascular cambia, depending on cell division, expansion and differentiation and resulting in a rapid increase in root diameter (Fig. 2). Therefore, detailed information on gene expression is vital to understand the molecular mechanism of any developmental process. Although *C. speciosa* has great economic value, genetic information for this species remains unavailable. Here, roots at four different key developmental ages of *C. speciosa* were analyzed using an integrated morphological, hormonal and transcriptomic approach. A total of 4337 genes were identified that have shown differential expression during root thickening. Large numbers of DEGs were involved in similar molecular processes during the development of tuberous roots (Fig. 3), a dynamic process controlled by plant hormones, TFs and metabolism regulation pathways^20^.

### Hormonal signaling regulation during root thickening

The initiation and development of tuberous roots have been shown to be regulated by endogenous phytohormones^21^. The synergistic actions of various phytohormones, such as auxins, ABA, GAs, ETH, JA and CKs, finally result in the bulking of tuberous roots/stems^10,11^. The present study revealed that significant accumulation of JA appeared at the initial development of tuberous roots, but its content showed gradual decrease during storage root development (Fig. 4a). Previous studies have showed that JA might induce storage root formation by stimulating proliferation of metaxylem and cambium cells in sweet potato^22^. Thus, the high accumulation of JA might play an important role in the initial development of tuberous roots in *C. speciosa*. Jasmonate zinc finger inflorescence meristem (ZIM)-domain (JAZ) protein and JA receptor COI1 were reported to form COI1–JAZ complex in JA signaling in cells for the bioactive JA-ILE, subsequently JAZ proteins were degraded after being transferred to the 26S proteasome, and simultaneously, TFs were released to activate the expression of downstream genes^23^. The genes encoding JA ZIM-domain protein (*TIFYs*) and receptor *COIs* were highly expressed at the initial thickening stage (6 MAG) in the present study (Fig. 4b), which let us speculate that the COI1–JAZ complex might be related to JA mobilization of reserve metabolism and promotion of root thickening and isoflavonoid biosynthesis.

Auxins are a general coordinator of growth and development, and play important roles in tuberization and stress resistance^21^, as well as proliferation of cambium cells, and cell expansion^22^. Our results showed that the IAA content at the rapid thickening of tuberous roots (12 and 18 MAG) was higher than that at other age points, suggesting that high IAA levels might promote tuber enlargement (Fig. 4a). Among three primary auxin-induced gene families (*GH3*, *Aux/IAA* and *SAUR*), *GH3* gene families have been shown to be involved in root development. Most transcripts encoding *Aux/IAA* genes responsible for cambium secondary differentiation during radish taproot thickening were down-regulated, while those encoding *ARFs* genes and two *SAUR* transcripts were up-regulated at the cortex splitting and expanding stage^24^. Researchers suggested that the endogenous IAA content and genes involved in auxins signaling pathway, including *Aux/IAAs* and *ARFs* were highly expressed in expansion stage^20,25^. Congruent with previous studies, 6 *Aux/IAAs*, 4 *SAURs,* 2 *ARFs* and 2 *CH3s*, were highly expressed at the rapid thickening stage (12 and 18 MAG) in this study (Fig. 4b), implying that they might associate with the cell expansion.

Interestingly, the endogenous GA_3_ content and the expression of some genes related to the GA signaling pathway were significantly enhanced at the rapid-thickening stages, such as *GIDs*, *SCLs* and *CIGR2* in the present study (Fig. 4). In general, GAs are thought to play different roles in storage root formation and development. In potato, GAs were important promoters in stolon initiation but served as inhibitors of tuber initiation^26^. Gene encoding GID1-like gibberellin receptor (*StGID1*) was significantly up-regulated in the sample pair potato grafted with tomato (St-R) vs self-grafted potato (St-WT) during stolon development, which was consistent with GA_3_ content^12^. In addition, ABA and GAs were reported to antagonistically mediate tuberization in several species of plants, such as potato^11^, sweet potato^22^ and *P. notoginseng*^20^. The GA_3_ levels increased at the rapid-thickening stages of tuberous roots (12 and 18 MAG), whereas the ABA content showed gradual increase along with the process of tuberous root development in this study (Fig. 4a). Therefore, we hypothesized that antagonistic interaction between ABA and GA signaling potentially modulated root thickening in *C. speciosa*; this supposition was supported by the expression levels of genes related to ABA and GA signal transduction pathway in roots (Fig. 4).

In sweet potato, CKs appeared to be key factors in the formation of storage roots as a prerequirement for cambial cell proliferation^27^. In this study, *cis*-zeatin (*c*Z) was the main cytokinin in *C. speciosa* tuberous roots, and the peak of *c*Z content was at the mid-rapid thickening stage (18 MAG) (Fig. 4a). Overall, our result suggested that various phytohormones play roles at different ages of storage root development, which were supported by the previous studies in sweet potato^10^, radish^24^ and yam^25^. Nevertheless, the synergistic effect of phytohormones on *C. speciosa* tuberous root formation and development warrants further investigation.

### Starch and sucrose metabolism regulation during root thickening

We found that “starch and sucrose metabolism” pathway was markedly enriched in carbohydrate metabolism in *C. speciosa* (Supplementary Fig. S1f), which was consistent with the starch accumulation in the tuberous roots (Fig. 1b). Starch is an important carbohydrate that stored in tuberous roots. The accumulation of starch includes the synthesis, degradation, transport, and conversion of sucrose^28^. The main photoassimilate sucrose is a promoter that can stimulate the formation of tuberous roots in several plants^29^. In the starch and sucrose metabolic pathway, sucrose is converted into UDP-glucose and fructose by sucrose synthase, meanwhile it’s also converted to glucose and fructose by invertase in the cytosol^29^. Here, genes encoding ten enzymes in the starch and sucrose metabolic pathway were identified (Fig. 5). Genes related to sucrose metabolism were highly expressed at the rapid thickening stages, e.g., *SUS2*, *SUS-like*, *HXK1*, *GPI* (Fig. 5; Supplementary Table S3), suggesting that sucrose catabolism is necessary for root rapid-thickening in *C. speciosa.* Similar results were obtained in *Panax notoginseng*^20^, *Cyperus esculentus*^30^, and radish^31^. Glucan endo-1,3-beta-glucosidase (elgC) is widely distributed in higher plants, which is responsible for hydrolysis of the glycosidic bond in specific polysaccharides with tracts of unsubstituted β-1,3-linked glucosyl residues to release glucose^32^. Our study showed that five transcripts encoding eglC were highly expressed at the rapid thickening stages (Fig. 5), which means that they might contribute to glucose supply during root thickening process.

Several enzymes have been reported to be pivotal in starch biosynthesis and composition, including GBSS, SBE, and PGM^20^. Our transcriptome data showed that *GBSS1* was highly expressed at the stable thickening stage (30 MAG) (Fig. 5), and it means that starch accumulation in an active sink might play a vital role at the stable thickening stage, which was similar to the result in *P. notoginseng*^20^. The activity of beta-amylase (BAMY) could decrease by silencing *StBAM1* and *StBAM9* in potato, and *StBAM9* interacted with *StBAM1* on the starch granules^33^. Genes encoding BAMY were highly expressed at the expansion stage of yam tuberous roots^25^. In this study, *BAM9* was highly expressed at the mid-rapid thickening stage (18 MAG), whereas *BAM1* had a higher expression at 6 MAG (Fig. 5; Supplementary Table S3), implying that they might associate with starch granules. These starch and sucrose metabolism genes were required for the root thickening in *C. speciosa*, which might play vitol roles in the thickening of *C. speciosa* tuberous roots.

### Potential DEGs involved in the isoflavonoid biosynthetic pathway

The benefits of isoflavonoid compounds in plants have inspired efforts to identify the structural and regulatory genes in their biosynthetic pathway. Most of the isoflavonoid pathway genes encoding the key enzymes responsible for its biosynthesis have been identified^17^. Chalcone established the first step in the branched pathway for the synthesis of flavonoids, which was catalyzed by CHS^34^. It was reported that 9-member CHS family in soybean, in which *CHS7* and *CHS8* genes played critical roles in regulation of isoflavonoid biosynthesis^35^. In this study, *CHS1* and *CHS7* were significantly up-regulated at the rapid thickening stage of tuberous roots (Fig. 6), in which formononetin and maackiain was rapidly accumulated (Fig. 1). *CHIs* might regulate the efficient metabolic flux of isoflavonoid biosynthesis by interacting with *CHS1*, *IFS1*, and *CYP93C* in soybean^36^. It was reported that genes encoding CHI, CHS, CYP, and isoflavone reductase (IFR) were all involved in the biosynthesis pathway of bioactive isoflavonoids and most abundantly expressed in the summer-collected tubers in *Pueraria mirifica*^37^. Many isoflavonoids could also be *O*-methylated at hydroxyl groups by *O*-methyltransferases (OMTs), and 4′-*O*-methylated under the action of HI4′OMTs, which was reported to be important for isoflavonoids (e.g., formononetin) biosynthesis in several leguminous plant species^38^. The key step of pterocarpan phytoalexin (e.g., maackiain) biosynthesis, conversion of vestitone to pterocarpans, proved to be catalyzed by VR^17^. Our results showed that the expression of some genes (e.g., *HIDM-3*, *IFSs*, *I3′H*, *VRs*, and *HI4′OMT*) was at lower levels at 12 MAG in the present study (Fig. 6), which allowed us to speculate that the tailoring processes, including hydroxylation, methylation, and glycosylation, might be suppressed at the early-rapid thickening stage of *C. speciosa*. Several members of CYP81E subfamily, which catalyzed the hydroxylation of isoflavones, daidzein and formononetin, were identified to characterize the isoflavonoid metabolism in *Glycyrrhiza echinata* (CYP81E1), *Medicago truncatula* (CYP81E7) and soybean (CYP81E11, CYP81E12 and CYP81E18)^17,39,40^. In the present study, the transcripts encoding I3′H, belonging to CYP81E subfamily, were highly expressed at the 6 and 30 MAG (Fig. 6). Therefore, all data suggested that these DEGs might be potentially involved in the isoflavonoid biosynthesis.

### Transcription factors regulation during root thickening

Previous studies have revealed that TFs were important regulators of tuberous root formation and secondary metabolism, such as AP2/ERF, MYB, MYC, and WRKY^5,20^. In the current study, a total of 230 TF-encoding genes belonging to 30 TF families were identified from 4337 DEGs during root thickening. Among them, 41 TF genes, belonging to AP2/ERF, MYB, NAC, MADS, bHLH, and WRKY families, have previously implicated in plant growth and development (Supplementary Fig. S5). In *Arabidopsis*, *ERF109* was reported to positively control root stem cell niche maintenance and root growth through phytosulfokine (PSK) peptide hormones^41^, and mediate the cross-talk between JA signaling and IAA biosynthesis to regulate root development^42^. In this study, a gene homologous to *AtERF109* was highly expressed at early-rapid thickening stage (Supplementary Fig. S5). Recent studies have further demonstrated that JA-responsive ERF109 transcriptional regulation initiated the transition from seed roots to taproot thickening in *P. notoginseng*^20^. It’s unsurprising that endogenous JA content was dominantly increased in the early stage of tuberous root development and genes related to JA signaling were at high expression levels at the same time (Fig. 4), indicating that JA-induced ERF transcriptional regulation controled root thickening in *C. speciosa*. Moreover, several transcription factors including MYB (*MYB44s*), bHLH (*bHLH35* and *MYC2*), WRKY (e.g., *WRKY41*), and NAC (e.g., *NAC2*) were highly expressed at the rapid thickening stages (Supplementary Fig. S5). The bHLH transcription factors *MYC3* and *MYC4* were targets of JAZ repressors and acted additively with *MYC2* in the activation of jasmonate responses^43^. *MYB44*, a tuber-specific and sucrose-inducible element-binding factor in potato^44^, was reported to positively regulate the enhanced elongation of primary roots by inducing *N*-3-oxo-hexanoyl-homoserine lactone^45^ and negatively regulate anthocyanin biosynthesis at high temperatures in tuber flesh of potato^46^. An increasing number of data indicated that some WRKY genes (e.g., *WRKY41*) were responsive to phytohormones, such as ABA, GA and JA, to regulate the lateral root formation^47–49^. NAC TF family was reported to function in regulating secondary xylem development, secondary cell wall metabolism and cell expansion^50^. Furthermore, some up-regulated transcripts in this study, e.g., *MYB44s*, *bHLH35*, and *WRKY41*, which were reported to regulate the anthocyanin biosynthesis^46,51,52^, might play vital roles in the regulating of isoflavonoid biosynthesis. All these studies suggested that these TF genes might be potentially involved in the root thickening and isoflavonoid metabolism of *C. speciosa*.

### Regulatory model associated with tuber thickening and isoflavonoid biosynthesis of *C. speciosa*

**Figure 8:**
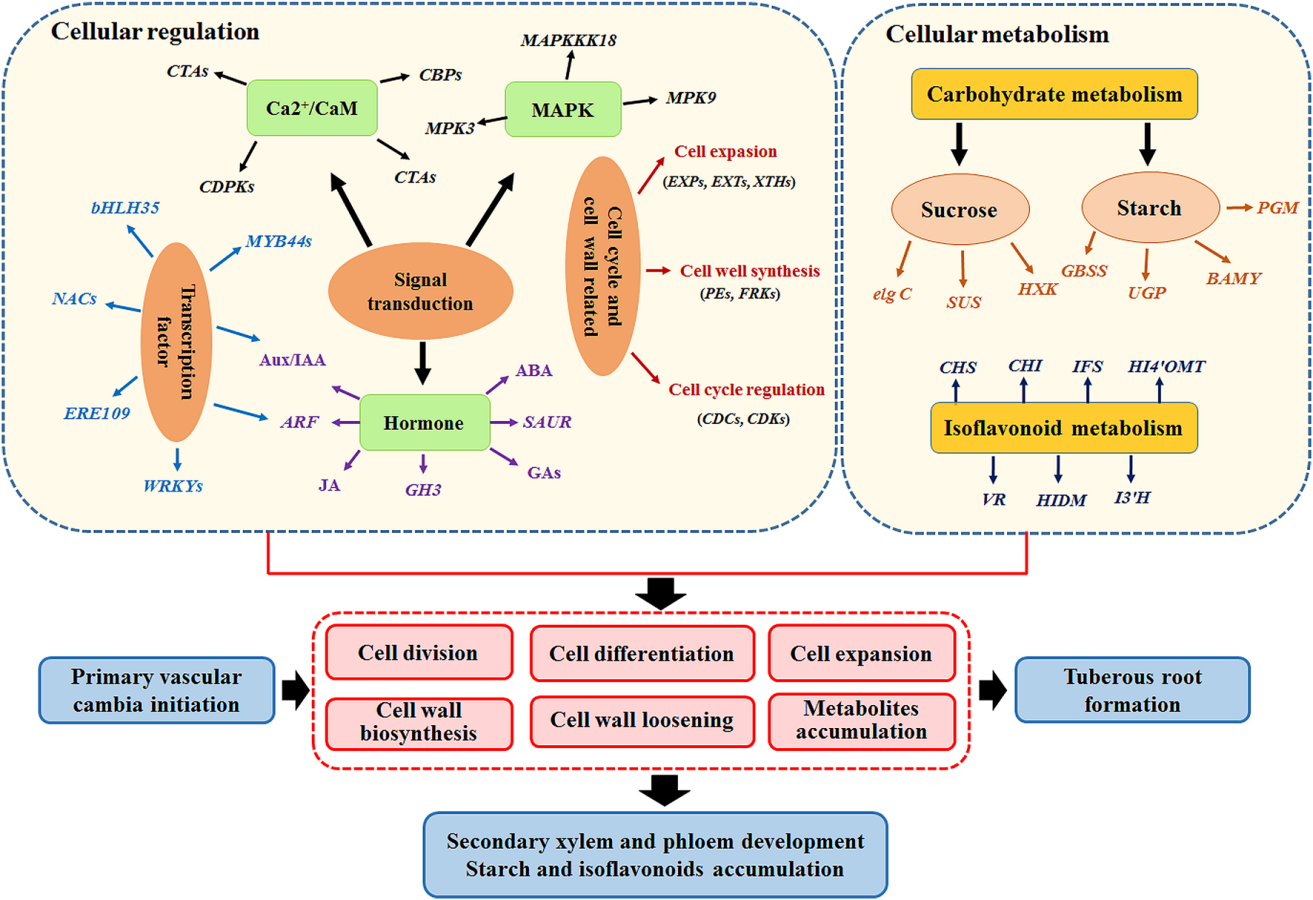
A proposed model of genetic regulatory mechanism during root thickening of *C. speciosa*.

The initiation and development of storage roots is a highly complicated process that multiple pathways regulate the development of secondary anomalous cambia and the accumulation of starch, sucrose and other chemical components^10^. In this study, a potential regulatory model associated with the root thickening and isoflavonoid biosynthesis of *C. speciosa* was proposed according to our integrated phenotypic and transcriptomic data (Fig. 8), which would help in understanding the underlying mechanisms of tuberous root formation and isoflavonoid biosynthesis in *C. speciosa*. Cell divisions and expansions in the vascular cambia, together with differentiation of secondary xylem and phloem under the activity of vascular cambia, leaded to an expansion of roots. The cell proliferation through several signal transduction pathways (calcium, MAPK, hormone and transcription signaling) and metabolism possesses (cell wall, sucrose, starch and isoflavonoid metabolism). Several genes were highly expressed to promote cell differentiation, division, expansion and dry matter accumulation at the secondary structure. In detail, *CBPs*, *CBLs*, *CDPKs*, *NCLs*, and *CTAs* were involved in calcium signaling pathway, a primary essential signaling transduction category required for normal growth and development of plants^53,54^. The MAPK signal pathway was reported to play a role in regulating the hormone signaling and developmental processes^5,54^, and four MAPK signaling-related genes were differentially expressed, including *MPK3*, *MPK7*, *MPK9* and *MAPKKK18*. *EXPs*, *EXTs*, *XTHs*, *PEs*, *CDCs*, *CDKs*, and *FRKs* were reported to regulate the cell extension, expansion, cell wall synthesis, and cell cycle regulation^5,20^. The hormone signaling are critical factors for regulating root tuberization and isoflavonoid metabolism at different developmental stages, including synergistic and antagonistic regulation^11,22,55^. Most genes involved in hormone signal transduction pathways were up-regulated at the rapid thickening stages of tuberous roots. JA might induce storage root formation by stimulating proliferation of metaxylem and cambium cells^22^. *MYB44s*, *ERF109*, *WRKYs*, *NACs* were involved in enhancing the formation and elongation of roots^20,45,48^. In addition, *MYB44s*, *bHLH35*, and *WRKY41* were also reported to regulate the anthocyanin biosynthesis^46,51,52^, which might be involved in isoflavonoid biosynthesis. Genes encoding GBSS, SUS, PGM, UGP, and BAMY, were reported to be associated with sucrose hydrolyzation and starch synthesis during tuberous root thickening in several species^5,20^, while genes encoding CHI, CHS, HIDM, HI4′OMT, VR, IFS and I3*′*H were found to be involved in the isoflavonoid metabolism pathway^17,35–37^. Taken together, the results indicated that all these DEGs potentially involved in the regulatory model of root thickening and isoflavonoid biosynthesis in *C. speciosa*. Further functional identification studies were needed to confirm these potential genes.

## Conclusions

Integrated morphological, hormonal and transcriptomic analyses were performed in the present study, and multiple tuberous root development and isoflavonoid biosynthesis associated events, including cambium development, endogenous hormones change and corresponding candidate genes were revealed. JA might be related to the initial development of tuberous roots. ABA seemed to be essential for tuber maturation, whereas IAA, *c*Z and GA_3_ were correlated to rapid thickening of tuberous roots. Functional analysis showed that 15 DEGs participated in isoflavonoid biosynthesis, while 153 DEGs that involved in starch/sucrose metabolism, hormonal signaling, transcriptional regulation and cell wall metabolism, were identified to potentially control root thickening. A hypothetical model of genetic regulation associated with root thickening and isoflavonoid biosynthesis in *C. speciosa* is proposed, which will be valuable for further research and will help in understanding the underlying mechanism of tuberous root formation and isoflavonoid biosynthesis in *C. speciosa*.

## Methods

### Plant material and growth condition

Seeds of *C. speciosa* (diploid) were incubated in wet perlite under dark conditions at 26 ± 2 ℃ until germination (approximately 4 days). Two days after germination, seedlings were cultured in a greenhouse equipped with a system for monitoring temperature (25 ± 4 °C), relative humidity (60 ± 5%) and natural photoperiod in the Guangxi Botanical Garden of Medicinal Plants, with the same cultivation measure. The voucher specimens were deposited in Guangxi Botanical Garden of Medicinal Plants (No. 12110401). Tuberous roots were collected at half-year intervals, including 6, 12, 18, 24, 30, 36 months after germination (MAG), and used for biological, anatomical and chemical analysis. Roots at four developmental ages (6, 12, 18, 30 MAG) were used for endogenous hormones and RNA-Seq analyses. Ten roots from ten plants were selected randomly from every biological replicate, except for thirty plants at 6 MAG. Then, the thickest section in the middle (5 cm long) of each root, was washed with distilled water, cut down into pieces, and mixed to establish a biological replicate. Each sampling point had three biological replicates. The samples used for transverse paraffin sections were cut into 2-mm-thick slices after collection and immediately fixed in 10 mL mixture [formalin: acetic acid: 70% alcohol (FAA) = 5:5:90] for more than 12 h, then stored at 70% alcohol. For RNA purification and hormone detection, samples were immediately frozen in liquid nitrogen after collection and stored at − 80 °C until further use. For content detection, the samples were dried in an oven at 60 ℃.

### Determination of growth indexes, starch and index compound content

The volume of fresh roots was calculated using the water displacement method. The fresh roots were weighed using an electronic balance, then dried to achieve constant weight in an oven at 80 ℃. Starch content was determined according to Firon et al.^13^. The content of formononetin and maackiain were detected following by Chen et al.^2^.

### Optical microscope analysis

After rinsed in ddH_2_O for three times, samples were dehydrated using an increasing ethanol series (70%, 80%, 85%, 90%, 95% and 100%, 1 h for each step), and permeabilized in turpentine oil. The method of paraffin section and optical microscope pictures photographed were performed as previously described^56^. Serial tissue sections (10-μm-thick) were stained with 70% aqueous safranin O followed by 95% fast green FCF in absolute alcohol.

### RNA library construction and transcriptome sequencing

Total RNA was isolated from roots by following the method described by Liu et al.^57^. For each sample, equal amount (20 μg) of total RNA (RIN > 8.0) was applied to construct cDNA library. Briefly, the Oligo (dT) selection was used to enrich the poly (A) mRNAs. The mRNA was fragmented and reversed transcription to double-strand cDNA (dscDNA) by N6 random primer. The synthesized cDNA was subjected to end-repair and then 3′ adenylated. Adaptors were ligated to the ends of these 3′ adenylated cDNA fragments. The ligation products were purified and several rounds of PCR amplification were performed to enrich the purified cDNA template. The PCR product was denatured by heat and the single strand DNA was cyclized by splint oligo and DNA ligase. Finally, twelve libraries (three replicates per sample) were sequenced in pair-end (2 × 100 bp) format BGISEQ-500 sequencing platform, reads were processed and de novo assembled, and the results were analyzed by the BGI Tech Solutions Co., Ltd. (BGI Tech) (BGI, Shenzhen, P. R. China).

### Transcriptome profiling and DEGs analysis

Adaptor sequences (reads with ambiguous bases ‘N’) and low-quality reads (reads having more than 20% of bases with quality ≤ 15) were filtered. The filtered clean reads (FASTQ formatted files) for samples can be accessed in the NCBI Sequence Read Archive (SRA) database (https://trace.ncbi.nlm.nih.gov/Traces/sra/) under the accession number of SUB6340191. Trinity software (v2.4.0) ^58^ was used to carry out the de novo assembly. Tgicl software (v2.0.6)^59^ was used to remove the redundant Trinity generated contigs. The unigenes were divided to two types, one type was cluster, which the prefix was CL with the cluster id behind it (in one cluster, there were several unigenes which similarity between them was more than 70%), another type was singleton, which the prefix was unigene. The resulting transcripts were then processed to retrieve associated GO items by BLAST2GO software (v2.5.0)^60^. Besides, functional databases NT, NR, KOG, KEGG, SwissProt, Pfam and InterPro were used to annotate genes function, while Blastn, Blastx, and InterProScan 5 were used to align genes. Differential expression of transcripts in different samples were also evaluated by fragments per kilobase of transcript per million mapped reads (FPKM) in order to normalize the calculation of gene expression. The differential expression of transcripts was compared by using the DEGseq R package (1.18.0)^61^, with a threshold of FPKM ≥ 10 in each pairwise comparison, |Log_2_FC (Fold Change)|≥ 1 and *p *value < 0.05. In addition, all the DEGs were mapped in GO and KEGG databases to perform GO enrichment and pathway analysis using GOseq R package. Enrichment analysis of KEGG pathways of DEGs was done by KOBAS software^62^. Multiple testing corrections were performed by controlling the false discovery rate (FDR) to less than 0.05. The enrichment analysis was tested by Fisher’s exact test. PlantTFDB (Plant Transcription Factor Database) was used to annotate TFs.

### qRT-PCR analysis

To validate the expression patterns of candidate genes in the different samples, qRT-PCR experiment was performed, as previously described^63^. The forward and reverse primers of sixteen randomly selected DEGs and reference gene were designed using Primer 5.0 (Supplementary Table S6) and synthesized by BGI-Shenzhen. Every candidate gene in each sample was conducted in three biological replicates with three technical replicates. Glyceraldehyde-3-phosphate dehydrogenase (GAPDH) was used as a reference gene. The relative expression of each candidate gene was calculated by the 2^−ΔΔCq^ formula^64^. The normalized values of relative expression and FPKM values were calculated using log10 fold variation measurements, and the correlation between RNA-seq and qPCR results was analyzed using these values.

### Determination of endogenous hormone contents

Plant materials (120 mg fresh weight) were added to liquid nitrogen, ground into powder, and extracted with methanol/water (8/2) at 4 °C. The extract was centrifuged at 12000 *g* under 4 °C for 15 min. The supernatant was collected, evaporated to dryness under nitrogen gas stream, then reconstituted in methanol/water (3/7). After centrifuged, the supernatant was collected for UPLC-MS/MS analysis to determine phytohormones content, including auxin, CKs, GAs, JA, and ABA, which were performed as described by Pan et al.^65^.

### Statistical analyses

Three repetitions were performed to determine each value and their standard deviations (SD). Data were analyzed using IBM SPSS Statistics 19.0 software (Ehningen, Germany) and presented as the means ± SD. The statistical significance was determined using Duncan’s multiple range test. Values in figures marked with different lowercase were significantly different at 0.05 probability levels.

## Supplementary information

Supplementary Figures.

Supplementary Table S1.

Supplementary Table S2.

Supplementary Table S3.

Supplementary Table S4.

Supplementary Table S5.

Supplementary Table S6.

## Data Availability

The datasets generated for this study can be found in the NCBI SRA repository, https://submit.ncbi.nlm.nih.gov/subs/sra/, with the GenBank accession no.: SUB6340191.

## Abbreviations

AP2/ERF: APETALA2/ethylene-responsive factor
BGLU: Beta-glucosidase
bHLH: Basic helix loop-helix transcription factor
CK: Cytokinin
GBSS: Granule-bound starch synthase genes
HI4′OMT: Isoflavone 4′-*O*-methyltransferase
IAA: Indole-3-acetic acid
KEGG: Kyoto encyclopedia of genes and genomes
PAL: Phenylalanine ammonialyase
NAC: NAM, ATAF, and CUC transcription factors
SS: Starch synthase
TPS: Trehalose-phosphate synthase
UGP: UTP-glucose-1-phosphate uridylyltransferase
