# Supplementary material for: Hormonal and transcriptional analyses provides new insights into the molecular mechanisms underlying root thickening and isoflavonoid biosynthesis in Callerya speciosa (Champ. ex Benth.) Schot
Source: Sci Rep. 2021 Jan 8;11:9. doi: 10.1038/s41598-020-76633-x (PMC7794344; doi:10.1038/s41598-020-76633-x)
Supplement: Supplementary file 1 — Supplementary Figures. [file 41598_2020_76633_MOESM1_ESM.docx]

**Hormonal and Transcriptional Analyses Provides New Insights into the Molecular Mechanisms Underlying Root Thickening and Isoflavonoid Biosynthesis in *Callerya speciosa* (Champ. ex Benth.) Schot**

Shaochang Yao^1*^, Zuzai Lan^2^, Rongshao Huang^1^, Yong Tan^1^, Ding Huang^1^, Jinyuan Gu^1^, Chunliu Pan^2*^


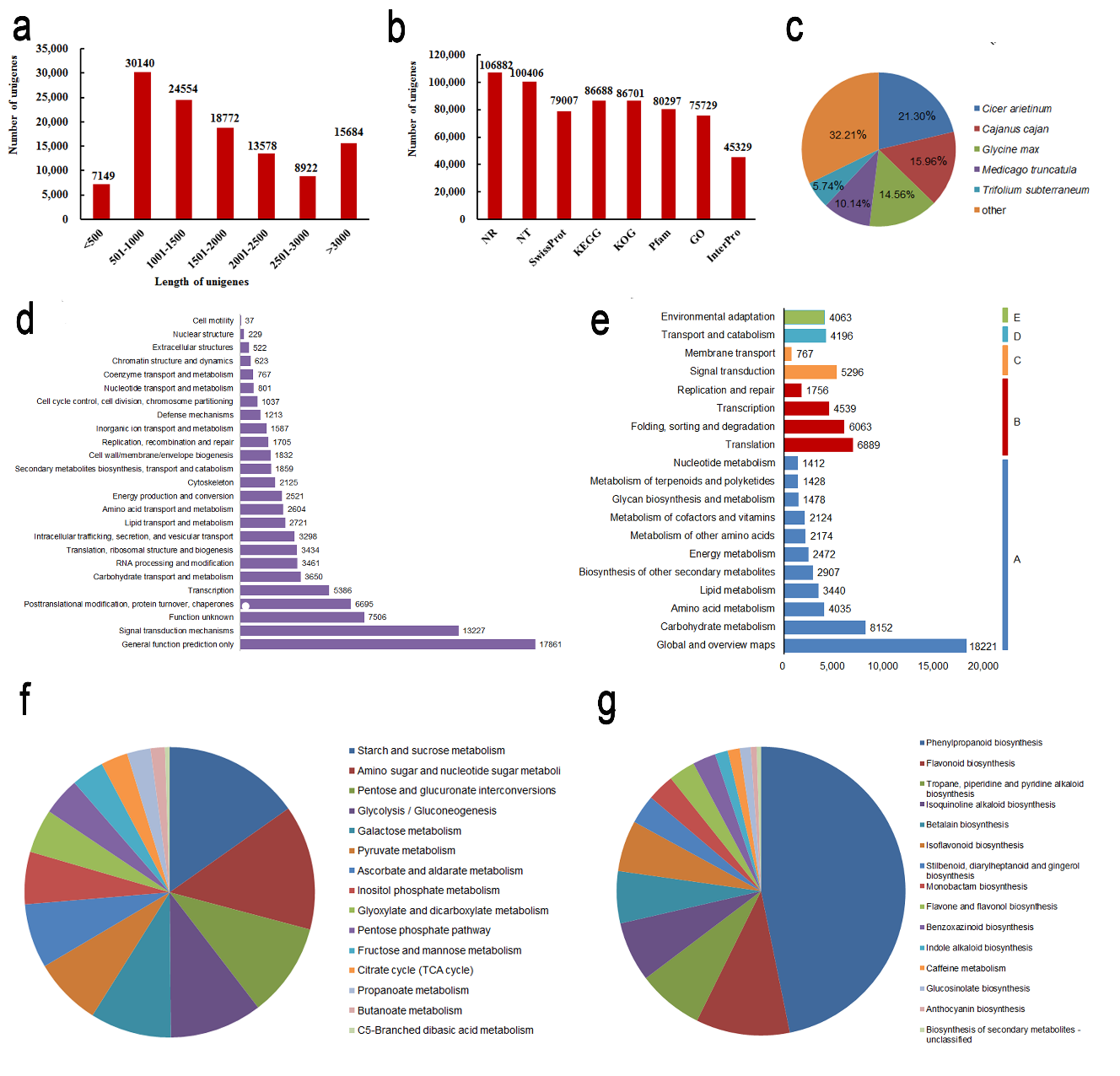


**Supplementary Fig. S1.** Overview of RNA-seq de novo transcriptome assembly and annotation. (a) Length distribution of assembled transcripts, the abscissa represents the length. (b) Number of unigenes aligned to different databases. (c) Distribution of species aligned by the assembled unigenes. (d) KOG annotation. (e) Functional distribution of KEGG annotation. e-A. Metabolism; e-B. Genetic Information Processing; e-C. Environmental Information Processing; e-D. Cellular Processes; e-E. Organismal Systems. (f) Sub-pathways of carbohydrate metabolism. (g) Sub-pathways of biosynthesis of other secondary metabolites.

**
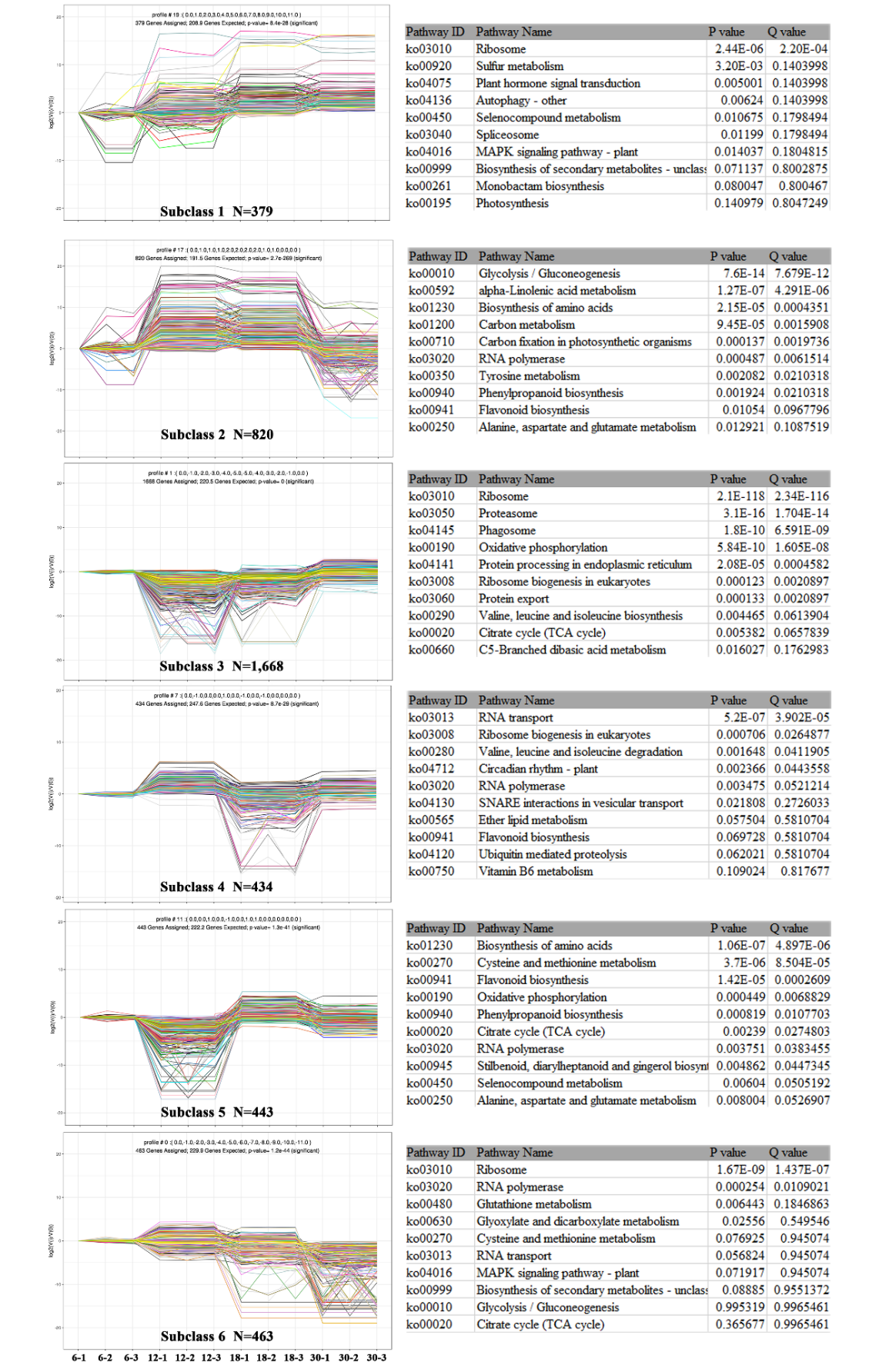
**

**Supplementary Fig. S2.** K-means clustering analysis of the DEGs and the top10 KEGG analysis. DEGs were mainly divided into eight subclasses. The numbers (6, 12, 18, 30) represent the four different developmental ages. All data shown reflected the results of three biological replicates (*n* = 3).


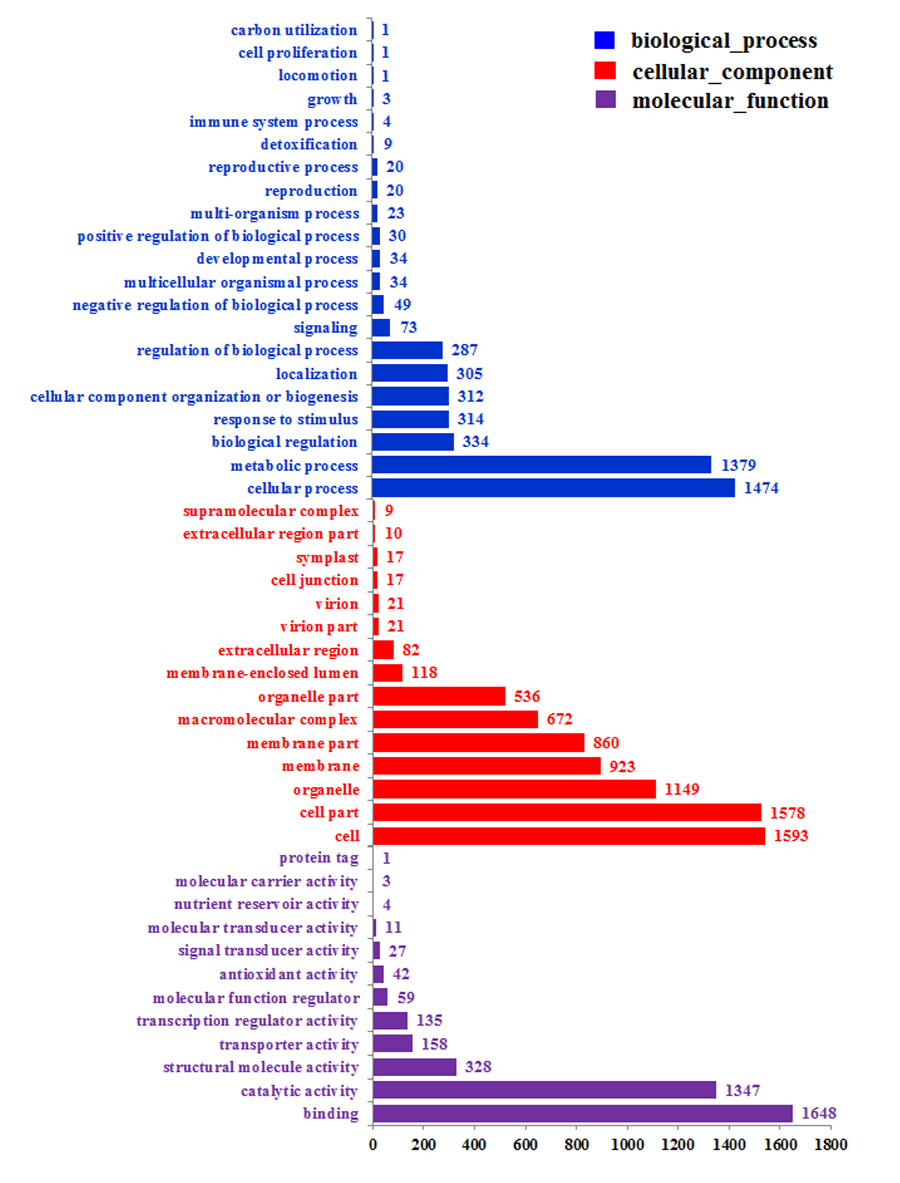


**Supplementary Fig. S3.** Gene Ontology (GO) function annotation of the 4,337 DEGs in *C. speciosa* roots.


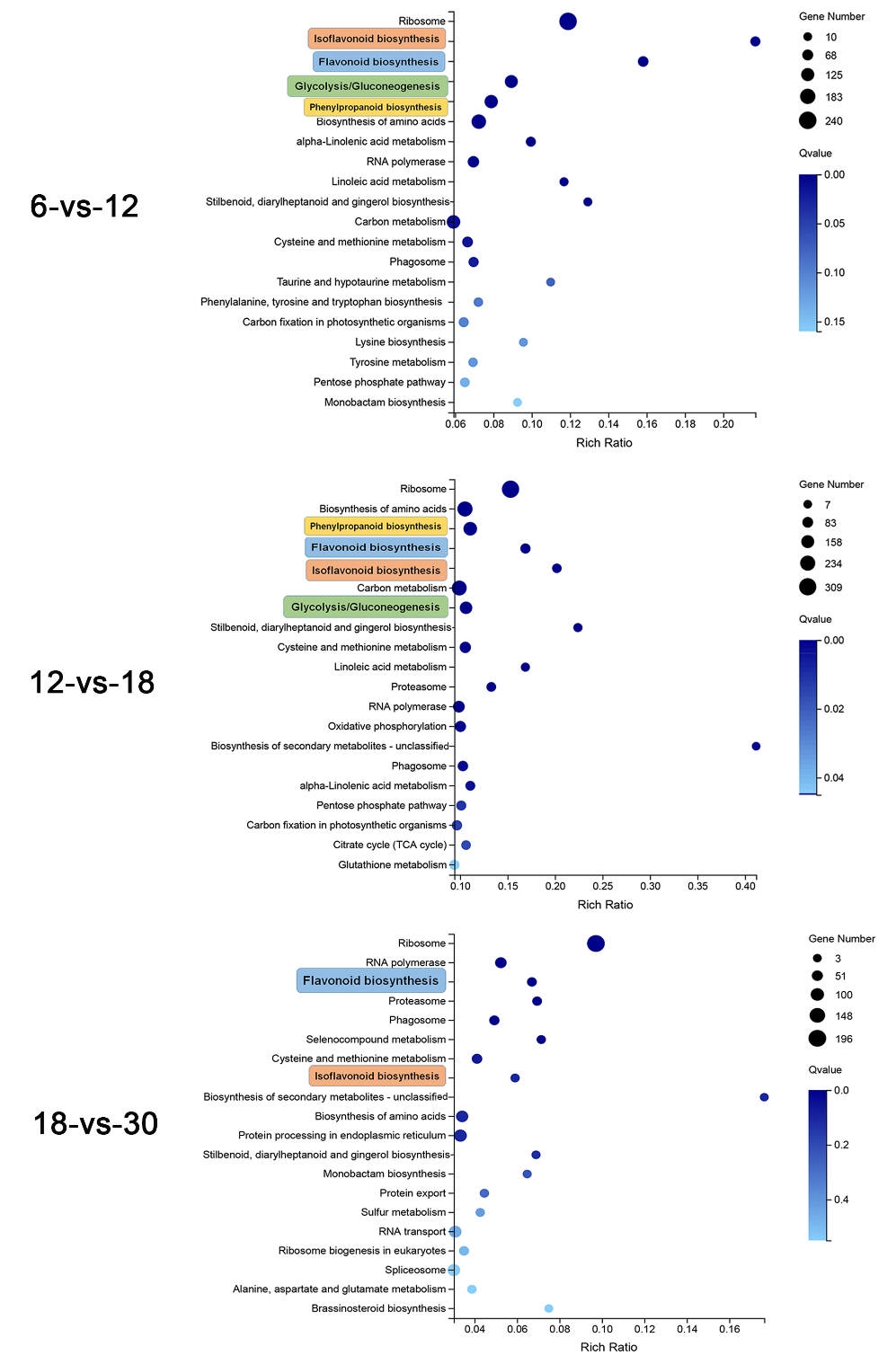


**Supplementary Fig. S4.** Significantly enriched KEGG pathways of DEGs. Top 20 significantly enriched KEGG pathways in three pairwise comparisons. The Y-axis on the left represented KEGG pathways, and the X-axis indicated the “enrich factor” represented by the ratio of DEGs numbers to total annotated gene numbers of each pathway. Lower q-values were shown darker in the blue circle. The area of a circle represented DEGs number. The “Phenylpropanoid biosynthesis”, “Flavonoid biosynthesis”, “Isoflavonoid biosynthesis”, and “Glycolysis/Gluconeogenesis” KEGG pathways colored with the yellow, blue, orange, and green bar, respectively.


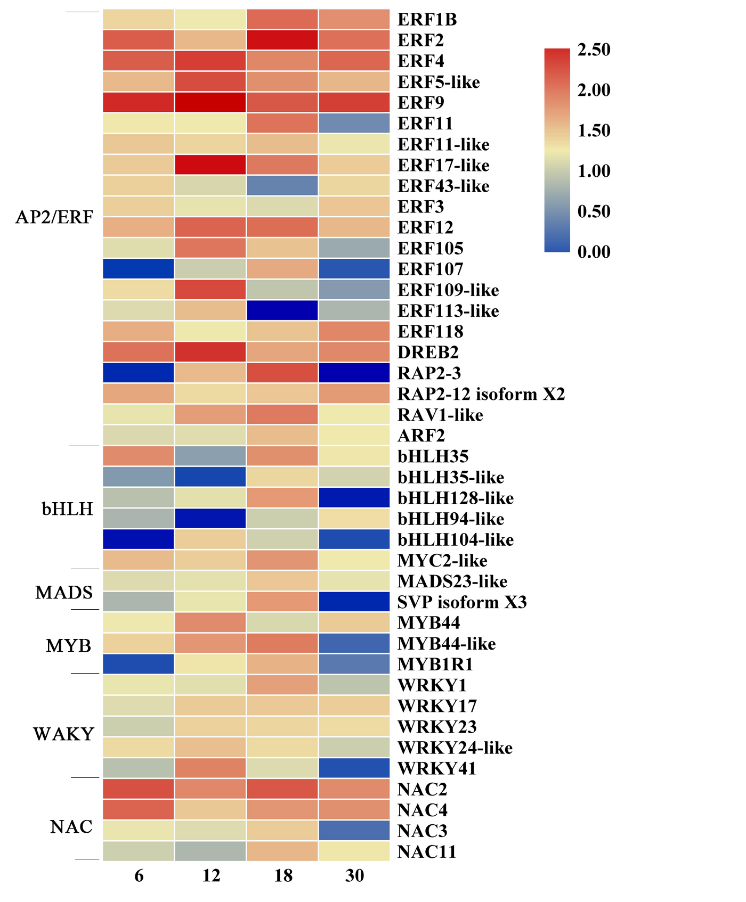


**Supplementary Fig. S5.** Expression profiles of DEGs encoding transcription factors at four age points. Heat map indicates the log_10_-transformed FPKM expression values. Changes in expression level were indicated by a change in color; from blue to red indicated an expression level from low to high.
